# A Novel Clustering Algorithm Inspired by Membrane Computing

**DOI:** 10.1155/2015/929471

**Published:** 2015-03-22

**Authors:** Hong Peng, Xiaohui Luo, Zhisheng Gao, Jun Wang, Zheng Pei

**Affiliations:** ^1^Center for Radio Administration and Technology Development, Xihua University, Chengdu 610039, China; ^2^School of Mathematics and Computer Engineering, Xihua University, Chengdu 610039, China; ^3^School of Electrical and Information Engineering, Xihua University, Chengdu 610039, China

## Abstract

P systems are a class of distributed parallel computing models; this paper presents a novel clustering algorithm, which is inspired from mechanism of a tissue-like P system with a loop structure of cells, called membrane clustering algorithm. The objects of the cells express the candidate centers of clusters and are evolved by the evolution rules. Based on the loop membrane structure, the communication rules realize a local neighborhood topology, which helps the coevolution of the objects and improves the diversity of objects in the system. The tissue-like P system can effectively search for the optimal partitioning with the help of its parallel computing advantage. The proposed clustering algorithm is evaluated on four artificial data sets and six real-life data sets. Experimental results show that the proposed clustering algorithm is superior or competitive to *k*-means algorithm and several evolutionary clustering algorithms recently reported in the literature.

## 1. Introduction

Data clustering is a fundamental conceptual problem in data mining, which describes the process of grouping data into classes or clusters such that the data in each cluster share a high degree of similarity while being very dissimilar to data from other clusters [1]. Over the past years, a large number of clustering algorithms have been proposed [2–4], which can be divided roughly in two categories: hierarchical and partitional. Hierarchical clustering proceeds successively by either merging smaller clusters into larger ones or splitting larger clusters. Partitional clustering attempts to directly decompose a data set into several disjointed clusters based on similarity measure, for example, mean square error (MSE). Clustering algorithms have been used in a wide variety of areas, such as pattern recognition, machine learning, image processing, and web mining [5, 6]. In the present study, *k*-means algorithm [7, 8] has received wide attention because of the following two reasons: (i) *k*-means has been recently elected and listed among the top most influential data mining algorithms [9] and (ii) it is at the same time very simple and quite scalable, as it has linear asymptotic running time with respect to any variable of the problem. However, *k*-means is sensitive to the initial centers and easy to get stuck at the local optimal solutions. Moreover, *k*-means takes large time cost to find the global optimal solution when the number of data points is large.

In recent years, some evolutionary algorithms have been introduced to overcome the shortcomings of *k*-means algorithm because of their global optimization capability. Several genetic algorithms- (GA-) based clustering algorithms have been proposed in the literature [10–14]. However, most of GA-based clustering algorithms can suffer from the degeneracy when numerous chromosomes represent the same solution. The degeneracy can lead to inefficient coverage of the search space as the same configurations of clusters are repeatedly explored. To overcome the shortcoming, particle swarm optimization- (PSO-) based or ant colony optimization- (ACO-) based clustering algorithms have been proposed. Kao et al. have proposed a hybrid technique based on combining the *k*-means and PSO for cluster analysis [15]. Shelokar et al. have introduced an evolutionary algorithm based on ACO for clustering problem [16]. Niknam and Amiri have presented a hybrid evolutionary optimization algorithm based on the combination of PSO and ACO for solving the clustering problem [17].

The aim of membrane computing is to abstract computing ideas (data structures, operations with data, ways to control operations, computing models, etc.) from the structure and the functioning of a single cell and from complexes of cells, such as tissues and organs including the brain. There are three main classes of P systems investigated: cell-like P systems (based on a cell-like (hence hierarchical) arrangement of membranes delimiting compartments where multisets of chemicals evolve according to given evolution rules) [18], tissue-like P systems (instead of hierarchical arrangement of membranes, consider arbitrary graphs as underlying structures, with membranes placed in the nodes while edges correspond to communication channels) [19], and neural-like P systems [20]. Many variants of all these systems have been considered, for example, [21, 22] for cell-like P systems, [23, 24] for tissue-like P systems, and [25–30] for neural-like P systems. An overview of the field can be found in [31], with up-to-date information available at the membrane computing website (http://ppage.psystems.eu/). These efforts have addressed the parallel computing advantage of P systems as well as the high effectiveness of solving a variety of difficult problems; especially, P systems can solve a number of NP-hard problems in linear or polynomial time complexity [32] and even solve PSPACE problems in a feasible time [33, 34]. Moreover, membrane algorithms have demonstrated a powerful global optimization performance [35–37].

This paper focuses on application of membrane computing to data clustering. Our motivation is applying the specially designed elements and inherent mechanisms of P systems to realize a novel clustering algorithm, called the membrane clustering algorithm.

## 2. Data Clustering Problem

Clustering is the process of recognizing natural groups or clusters from a data set based on some similarity measure. Suppose that data set *D* has *n* sample points, *x*_1_, *x*_2_,…, *x*_*n*_, *x*_*i*_ ∈ *R* ^*d*^ (*i* = 1,2,…, *n*), and is partitioned into *k* clusters, *C*_1_, *C*_2_,…, *C*_*k*_. Denote by *z*_1_, *z*_2_,…, *z*_*k*_ the corresponding centers. Usually, partitional clustering algorithm searches for the optimal centers in the solution space according to some clustering measure in order to solve data clustering problem. A commonly used clustering measure is

(1) $$\begin{array}{c} M\left(C_{1},C_{2},\ldots ,C_{k}\right)=\overset{n}{\underset{i=1}{\sum }}\;\overset{k}{\underset{j=1}{\sum }}w_{ij}\left\|x_{i}-z_{j}\right\|, \end{array}$$

where *w*_*ij*_ is the associate weight of point *x*_*i*_ with cluster* j*, which will be either 1 or 0 (if point *x*_*i*_ is allocated to cluster* j*, *w*_*ij*_ is 1, otherwise 0).

The clustering process, separating the objects into the clusters, is realized as an optimization problem. The goal of the optimization problem is to find the optimal centers by minimizing objective function 1:

(2) $$\begin{array}{c} \underset{z_{1},z_{2},\ldots ,z_{k}}{\min}J=\underset{z_{1},z_{2},\ldots ,z_{k}}{\min}M\left(C_{1},C_{2},\ldots ,C_{k}\right). \end{array}$$

In addition, the *M* value will be used to evaluate objects in the proposed clustering algorithm. If the *M* value of an object is the smaller one, the object is the better; otherwise, it is worse.

## 3. Proposed Membrane Clustering Algorithm

In this section the proposed membrane clustering algorithm is discussed in detail, which is inspired by the mechanism of membrane computing. A tissue-like P system with a loop structure of cells is designed as its optimization framework. The tissue-like P system with a loop structure of cells can be described as the following construct:

(3) $$\begin{array}{c} \Pi =\left(Z_{1},{\ldots ,Z}_{q},R_{1},{\ldots ,R}_{q},R^{\prime },i_{o}\right), \end{array}$$

where

(1) *Z*_*i*_ (1≤*i*≤*q*) is the set of *m* objects in cell *i*;
(2) *R*_*i*_ (1 ≤ *i* ≤ *q*) is the set of evolution rules in cell *i*, which contains three evolution rules: selection, crossover, and mutation rules;
(3) *R*′ is finite set of communication rules with the following forms:
  - antiport rule:  (*i*, *Z*/*Z*′, *j*), *i*, *j* = 1,2,…, *q*, *i* ≠ *j*. The rule is used to communicate the objects between a cell and its two adjacent cells;
  - symport rule:  (*i*, *Z*/*λ*, 0), *i* = 1,2,…, *q*. The rule is used to communicate the objects between cell and the environment.
(4) *i*_*o*_ indicates the output region of the system.

**Figure 1:**
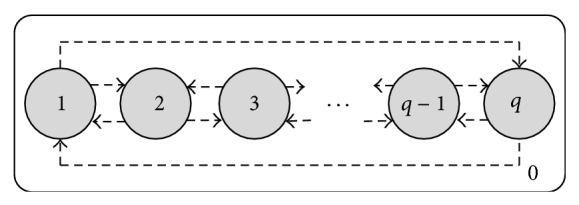
Membrane structure of the designed tissue-like P system.

Figure 1 shows membrane structure of the tissue-like P system, which consists of *q* cells. The *q* cells are labeled by 1,2,…, *q*, respectively. The region labeled by 0 is the environment and is also output region of the system. The directed lines in Figure 1 indicate the communication of objects between the *q* cells. Moreover, the *q* cells will be arranged as a loop topology based on the communication rules described below. As usual in P system, the *q* cells, as parallel computing units, will run independently. In addition, the environment always stores the best object found so far in the system. When the system halts, the object in the environment will be regarded as the output of the whole system.

The role of the tissue-like P system is to evolve the optimal centers of clusters for a data set; thus each object in cells will express a group of (candidate) centers. Thus, each object in cells is considered as a (*k* × *d*)-dimensional real vector of the form

(4) Z=z11,z12,…,z1d,…,zi1,zi2,0000…,zid,…,zk1,zk2,…,zkd,

where *z*_*i*1_, *z*_*i*2_,…, *z*_*id*_ are *d* components of *i*th cluster center *z*_*i*_, *i* = 1,2,…, *k*. For simplicity, suppose that each cell has the same number of objects, which is denoted by *m*.

Initially, the system will randomly generate *m* initial objects for each cell. When an initial object *Z* is generated, (*k* × *d*) random real numbers are produced repeatedly to form it with the constraint of

(5) $$\begin{array}{c} A_{1}\leq z_{i1}\leq B_{1},\ldots ,A_{j}\leq z_{ij}\leq B_{j},\ldots ,A_{d}\leq z_{id}\leq B_{d}, \end{array}$$

where *A*_*j*_ and *B*_*j*_ are lower bound and upper bound of* j*th dimensional component of data points, respectively, *j* = 1,2,…, *d*.

As usual, the tissue-like P system has two mechanisms: evolution and communication mechanisms. The two mechanisms will be described as follows.

### 3.1. Evolution Mechanism

**Figure 2:**
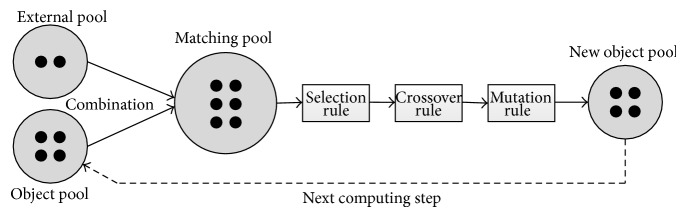
Evolution procedure of objects in a cell.

The role of evolution rules is to evolve the objects in cells to generate new objects used in next computing step. During the evolution, each cell maintains the same size (the number of objects). In this work, three known genetic operations (selection, crossover, and mutation) [38, 39] are used as the evolution rules in cells. In a computing step, all objects (located in object pool) in each cell and the best objects (located in external pool) from its two adjacent cells constitute a matching pool. The objects in external pool are actually the best objects communicated from its two adjacent cells in previous computing step. The objects in matching pool will be evolved by executing selection, crossover, and mutation operations in turn. In order to maintain the size of objects in each cell, truncation operation is used to constitute new object pool according to the *M* values of objects. The objects in new object pool will be regarded as the objects to be evolved in next computing step.  Figure 2 shows the evolution procedure of objects in a cell.

In this work, selection operation uses usual rotating wheel method, while crossover operation uses single-point crossover in which the position of crossover point is determined according to crossover probability *p*_*c*_ [39]. The single-point mutation is used to realize the mutations of objects. If *v* is a mutation point determined according to mutation probability *p*_*m*_, its value becomes, after mutating,

(6) $$\begin{array}{c} v^{\prime }=\left\{\begin{array}{cc} v\pm 2\delta v, & v\neq 0\\ v\pm 2\delta , & v=0, \end{array}\right. \end{array}$$

where the signs “+” or “−” occur with equal probability, and *δ* is real number in the range [0,1], generated with uniform distribution.

### 3.2. Communication Mechanism

The communication mechanism is used to exchange the objects between each cell and its two adjacent cells and update the best object found so far in the environment. The communication mechanism is realized by communication rules of two types: antiport rule (*i*, *Z*/*Z*′, *j*), which indicates that object *Z* is communicated from cell *i* to cell *j* and object *Z*′ is communicated from cell *j* to cell *i*, and symport rule (*i*, *Z*/*λ*, 0), which indicates that object *Z* is communicated from cell *i* to the environment.

**Figure 3:**
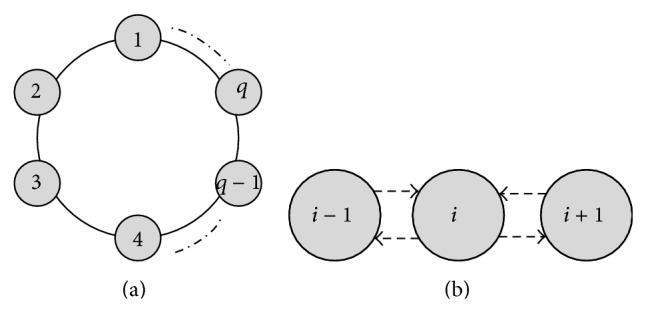
A loop topology structure of cells and the communication relation between adjacent cells.

The communication rules impliedly indicate the connection relationship between cells.  Figure 3 shows the communication relation of objects between cells in the designed tissue-like P system. From a logical point of view, the communication relation shows that the cells form a loop topology, shown in Figure 3(a). Meanwhile, this also reflects a neighborhood structure of the communication of objects; namely, each cell only exchanges and shares the objects with its two adjacent cells, shown in Figure 3(b). After the objects are evolved, each cell (such as cell *i*) transmits its several best objects into adjacent cells (such as cells *i* − 1 and *i* + 1) and retrieves several best objects from adjacent cells (such as cells *i* − 1 and *i* + 1) by using the communication rule, constituting the matching pool of objects in next computing step. The special logical structure can bring the following benefits.

1. The coevolution of objects in the *q* cells can accelerate the convergence of the proposed clustering algorithm.
2. The object sharing mechanism of the local neighborhood structure can enhance the diversity of objects in the entire system.

The communication of objects not only occurs between cells, but also appears between cell and the environment. The global best object found so far in whole system is stored always in the environment. After objects are evolved, each cell communicates its best object found in current computing step into the environment to update the global best object. The update strategy is that if *f*(*Z*) < *f*(*G*) then *G* = *Z*; otherwise, *G* retains unchanged, where *Z* is the current best object, *G* is the global best object, and *f*(·) is the fitness function (*M* value).

As usual in P system, the *q* cells, as parallel computing units, will run independently. In addition, the environment always stores the best object found so far in the system. In this work, maximum execution step number is used as the halting condition of the tissue-like P system; that is, the tissue-like P system will continue to run until it reaches the maximum execution step number. When the system halts, the object in the environment will be regarded as the output of whole system, namely, the found optimal centers.

Based on the tissue-like P system described above, the proposed membrane clustering algorithm is summarized in Algorithm 1.

## 4. Simulation Experiments

The proposed membrane clustering algorithm is evaluated on ten data sets and compared with classical *k*-means algorithm and several clustering algorithms based on evolutionary algorithms, including GA [10], PSO [15], and ACO [16]. In order to test the robustness of these clustering algorithms, we repeat the experiments 50 times for each data set.

**Figure 4:**
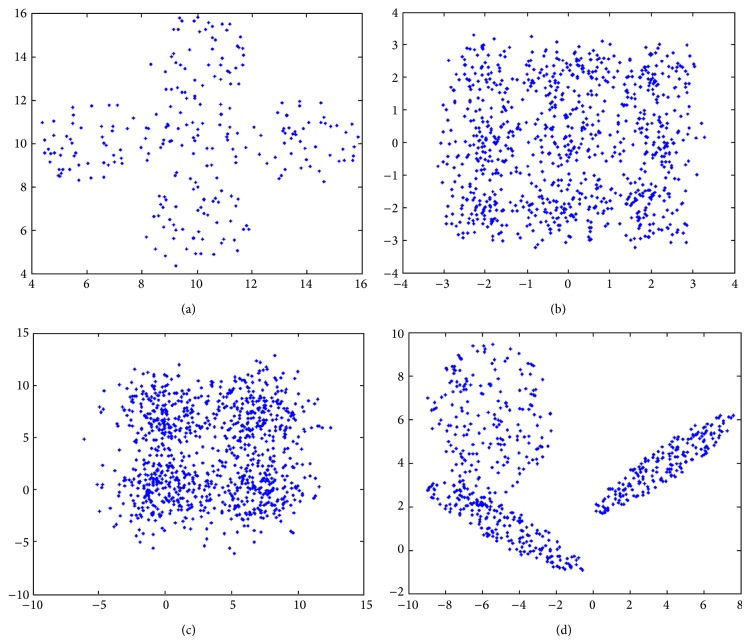
Four artificial data sets: (a)* AD_5_2*; (b)* Data_9_2*; (c)* Square_4*; (d)* Sym_3_22*.

In the experiments, two kinds of data sets are used to evaluate these clustering algorithms. First is the four manually generated data sets used in the existing literatures,* AD_5_2*,* Data_9_2*,* Square_4*, and* Sym_3_22*, shown in Figure 4. Second is the six real-life data sets provided in UCI [40], including the* Iris*,* BreastCancer*,* Newthyroid*,* LungCancer*,* Wine*, and* LiveDisorder*. The sizes of the data sets can be found in Table 1.

The proposed membrane clustering algorithm will be compared with *k*-means and three evolutionary clustering algorithms recently reported in the literature, including GA, PSO, and ACO. These algorithms are implemented in Matlab 7.1 according to the following parameters.

1. Tissue-like P systems. Each cell contains 100 objects and communicates its first five best objects into two adjacent cells. The maximum computing step number is chosen to be 200. In the implementation, evolution rules use the adaptive crossover probability *p*_*c*_ and mutation probability *p*_*m*_. In order to study performances of tissue-like P systems of different degrees, four cases are considered in the experiments: *q* = 4,8, 16,20.
2. GA [10]. In the rotating wheel method, single-point crossover and single-point mutation are used, where the crossover and mutation probabilities, *p*_*c*_ and *p*_*m*_, are chosen to be 0.8 and 0.001, respectively. Let the population size be *N*_swarm_ = 100 and let maximum iteration number be *t*_max⁡_ = 200.
3. PSO [15]. The *w* uses a linear decreasing inertia weight, where *w*_min⁡_ = 0.4 and *w*_max⁡_ = 0.9; *c*_1_ = *c*_2_ = 2.0, the population size NP = 100, and maximum iteration number is 200.
4. ACO [16]. The best parameter values are *γ*_1_ = *γ*_2_ = 1.0 and *ρ* = 0.99.

In the experiments, we realize four tissue-like P systems with degrees 4, 8, 16, and 20, respectively. The aim is to evaluate the effects of the number of cells (i.e., different degrees) on clustering quality. The four tissue-like P systems are applied to find out the optimal centers for the ten data sets, respectively. In this work, the *M* value is also used to measure the clustering quality of each clustering algorithm. Considering that the evolution rules in the designed tissue-like P system include stochastic mechanism, we independently execute the tissue-like P systems of the four degrees 50 times on each data set and then compute their mean values and standard deviations of the 50 runs. The mean values are used to illustrate the average performance of the algorithms while standard deviations indicate their robustness.  Table 2 provides experimental results of the tissue-like P systems of four degrees on ten data sets, respectively. The results of degrees 16 and 20 are better than those of the other two degrees, namely, lower mean values and smaller standard deviations. It can be further observed that the tissue-like P system with degree 16 obtains the smallest mean values and standard deviations on most of data sets. The results illustrate that the tissue-like P system with degree 16 has good clustering quality and high robustness.

In order to further evaluate clustering performance, the proposed membrane clustering algorithm is compared with GA-based, PSO-based, and ACO-based clustering algorithms as well as classical *k*-means algorithm.  Table 3 gives the comparison results of the tissue-like P system of degree 16 with other four clustering algorithms on the ten data sets, respectively. The comparison results show that the tissue-like P system provides the optimum average value and smallest standard deviation in comparison to those of other algorithms. For instance, the results obtained on the* AD_5_2* show that the tissue-like P system converges to the optimum of 326.4478 at almost times and PSO reaches to 326.44 in most of runs, while ACO, GA, and *k*-means attain 326.45, 322.31, and 332.47, respectively. The standard deviations of *M* values for the tissue-like P system, PSO, and ACO are 0.0105, 0.0128, and 0.0344, respectively, which are significantly smaller than the other two algorithms. For the results on the* Iris*, the optimum value is 96.75, which is obtained in most of runs of the tissue-like P system; however, the other four algorithms fail to attain the value even once within 50 runs. The results on the* Newthyroid* also show that the tissue-like P system provides the optimum value of 1869.29 while the PSO, ACO, GA, and *k*-means obtain 1872.51, 1872.56, 1875.11, and 1886.25, respectively. In addition, the tissue-like P system obtains smallest standard deviation on each data set in comparison to the other four algorithms, which illustrates that it has high robustness.

Wilcoxon's rank sum test is a nonparametric statistical significance test for independent samples. The statistical significance test has been conducted at the 5% significance level in the experiments. We create five groups for the ten data sets, which are corresponding to the five clustering algorithms (tissue-like P system, GA, PSO, ACO, and *k*-means), respectively. Each group consists of the *M* values produced by 50 consecutive runs of the corresponding algorithms. In order to illustrate if the goodness is statistically significant, we have completed a statistical significance test for these clustering algorithms.  Table 4 gives the *P* values provided by Wilcoxon's rank sum test for comparison of two groups (one group corresponding to the tissue-like P system and another group corresponding to some other method) at a time. The null hypothesis assumes that there is no significant difference between the mean values of two groups, whereas there is significant difference in the mean values of two groups for the alternative hypothesis. It is evident from Table 4 that all *P* values are less than 0.05 (5% significance level). This is a strong evidence against the null hypothesis, establishing significant superiority of the proposed membrane clustering algorithm.

## 5. Conclusion

In this paper, we discuss a membrane clustering algorithm, a novel clustering algorithm in the framework of membrane computing. Distinguished from the existing evolutionary clustering techniques, two inherent mechanisms of membrane computing are exploited to realize the membrane clustering algorithm, including evolution and communication mechanisms. For this purpose, a tissue-like P system consisting of *q* cells is designed, in which each cell as parallel computing unit runs in maximally parallel way and each object of the system represents a group of candidate centers. Moreover, the communication rules impliedly realize a local neighborhood structure; namely, each cell exchanges and shares the best objects with its two adjacent cells. Under the control of evolution and communication mechanisms of objects, the tissue-like P system is able to search for the optimal centers for a data set to be clustered. In addition, the local neighborhood structure can guide the exploitation of the optimal object and enhance the diversity of evolution objects.

## Figures and Tables

**Algorithm 1:**
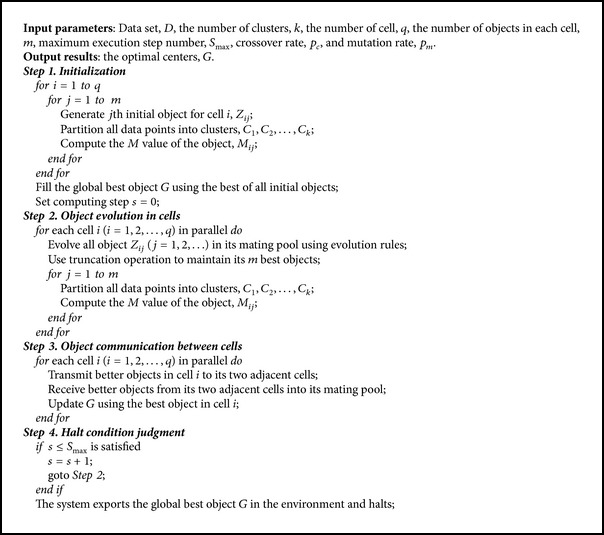
Membrane clustering algorithm: a clustering algorithm based on tissue-like P systems.

**Table 1:** Properties of the test data sets.

	Data	Input	Class
*AD_5_2 *	250	2	5
*Data_9_2 *	900	2	9
*Square_4 *	1000	2	4
*Sym_3_22 *	600	2	3
*Iris, *	150	4	3
*BreastCancer *	683	9	2
*Newthyroid *	215	5	3
*LungCancer *	32	56	3
*Wine *	178	13	3
*LiveDisorder *	345	6	2

**Table 2:** The performance comparisons of tissue-like P systems of different degrees.

Data set	4 cells	8 cells	16 cells	20 cells
*AD_5_2 *	327.01 ± 0.0944	326.94 ± 0.0277	326.44 ± 0.0105	326.94 ± 0.0312
*Data_9_2 *	591.11 ± 0.1331	591.12 ± 0.0510	591.06 ± 0.0280	591.03 ± 0.0537
*Square_4 *	2380.25 ± 0.1334	2380.26 ± 0.0956	2379.74 ± 0.0189	2380.00 ± 0.0729
*Sym_3_22 *	1248.31 ± 0.3156	1248.11 ± 0.0554	1247.72 ± 0.0105	1248.05 ± 0.0333
*Iris *	96.84 ± 0.0751	96.81 ± 0.0435	96.75 ± 0.0428	96.77 ± 0.0361
*BreastCancer *	2974.24 ± 1.5431	2971.14 ± 1.5287	2970.24 ± 1.1225	2969.06 ± 1.0970
*Newthyroid *	1885.69 ± 14.377	1870.37 ± 1.7355	1869.29 ± 0.9215	1871.18 ± 2.2496
*LungCancer *	124.69 ± 0.0045	124.69 ± 0.0012	124.69 ± 0.0011	124.69 ± 0.0035
*Wine *	16309.01 ± 2.5053	16303.42 ± 1.9595	16292.25 ± 0.1529	16301.97 ± 2.8563
*LiveDisorder *	9860.54 ± 5.7239	9859.02 ± 0.5116	9851.78 ± 0.0347	9857.08 ± 0.1043

**Table 3:** The results obtained by the algorithms for 50 runs on the ten data sets.

Data set	P systems	GA	PSO	ACO	*k*-means
*AD_5_2 *	326.44 ± 0.0105	332.31 ± 0.4792	326.44 ± 0.0128	326.45 ± 0.0344	332.47 ± 3.1286
*Data_9_2 *	591.06 ± 0.0280	593.72 ± 0.2635	591.14 ± 0.0303	591.42 ± 0.0372	623.57 ± 3.1326
*Square_4 *	2379.74 ± 0.0189	2380.33 ± 0.6319	2379.74 ± 0.0226	2379.79 ± 0.0428	2386.00 ± 4.5217
*Sym_3_22 *	1247.72 ± 0.0105	1249.36 ± 1.2163	1247.72 ± 0.0149	1247.75 ± 0.0315	1255.45 ± 3.8725
*Iris *	96.75 ± 0.0428	99.83 ± 5.5239	97.23 ± 0.3513	97.25 ± 0.4152	104.11 ± 12.4563
*BreastCancer *	2970.24 ± 1.1225	3249.26 ± 229.734	3050.04 ± 110.801	3046.06 ± 90.500	3251.21 ± 251.143
*Newthyroid *	1869.29 ± 0.9215	1875.11 ± 13.5834	1872.51 ± 11.0923	1872.56 ± 11.1045	1886.25 ± 16.2189
*LungCancer *	124.69 ± 0.0011	129.52 ± 4.4961	127.23 ± 1.1528	127.31 ± 1.2936	139.40 ± 7.3136
*Wine *	16292.25 ± 0.1529	16298.42 ± 2.1523	16292.25 ± 0.1531	16292.25 ± 0.1672	16312.43 ± 9.4269
*LiveDisorder *	9851.73 ± 0.0347	9856.14 ± 1.9523	9851.73 ± 0.0356	9851.74 ± 0.0692	9868.32 ± 7.9274

**Table 4:** The results of *P* values produced by Wilcoxon's rank sum test.

P systems	GA	PSO	ACO	*k*-means
*AD_5_2 *	4.1321*e* − 3	2.3256*e* − 2	2.6351*e* − 2	3.4273*e* − 3
*Data_9_2 *	4.0536*e* − 3	2.2734*e* − 2	2.7932*e* − 2	3.2963*e* − 3
*Square_4 *	3.9275*e* − 3	2.1482*e* − 2	2.8175*e* − 2	3.5387*e* − 3
*Sym_3_22 *	3.7894*e* − 3	2.4357*e* − 2	2.8529*e* − 2	3.4416*e* − 3
*Iris *	4.0968*e* − 3	3.5823*e* − 2	3.2634*e* − 2	3.6528*e* − 3
*BreastCancer *	3.9235*e* − 3	2.9527*e* − 2	2.8192*e* − 2	3.4632*e* − 3
*Newthyroid *	3.8864*e* − 3	2.5162*e* − 2	2.9355*e* − 2	3.5381*e* − 3
*LungCancer *	3.8575*e* − 3	2.7346*e* − 2	2.7358*e* − 2	3.5138*e* − 3
*Wine *	3.7639*e* − 3	3.2189*e* − 2	2.7963*e* − 2	3.6348*e* − 3
*LiveDisorder *	3.8398*e* − 3	2.4671*e* − 2	2.8846*e* − 2	3.5822*e* − 3

## References

[1] Hartigan J. A. (1975). *Clustering Algorithms*.

[2] Jain A. K., Dubes R. C. (1988). *Algorithms for Clustering Data*.

[3] Xu R., Wunsch D. (2005). Survey of clustering algorithms. *IEEE Transactions on Neural Networks*.

[4] Jain A. K. (2010). Data clustering: 50 years beyond K-means. *Pattern Recognition Letters*.

[5] Everitt B., Landau S., Leese M. (2001). *Cluster Analysis*.

[6] Saha S., Bandyopadhyay S. (2010). A symmetry based multiobjective clustering technique for automatic evolution of clusters. *Pattern Recognition*.

[7] Kanungo T., Mount D. M., Netanyahu N. S., Piatko C. D., Silverman R., Wu A. Y. (2002). An efficient k-means clustering algorithms: analysis and implementation. *IEEE Transactions on Pattern Analysis and Machine Intelligence*.

[8] Steinley D. (2006). *K*-means clustering: a half-century synthesis. *The British Journal of Mathematical and Statistical Psychology*.

[9] Wu X. (2009). *Top Ten Algorithms in Data Mining*.

[10] Bandyopadhyay S., Maulik U. (2002). An evolutionary technique based on K-means algorithm for optimal clustering in ℝ^*N*^. *Information Sciences*.

[11] Bandyopadhyay S., Saha S. (2007). GAPS: a clustering method using a new point symmetry-based distance measure. *Pattern Recognition*.

[12] Laszlo M., Mukherjee S. (2007). A genetic algorithm that exchanges neighboring centers for *k*-means clustering. *Pattern Recognition Letters*.

[13] Chang D. X., Zhang X. D., Zheng C. W. (2009). A genetic algorithm with gene rearrangement for K-means clustering. *Pattern Recognition*.

[14] Nguyen C. D., Cios K. J. (2008). GAKREM: a novel hybrid clustering algorithm. *Information Sciences*.

[15] Kao Y. T., Zahara E., Kao I. W. (2008). A hybridized approach to data clustering. *Expert Systems with Applications*.

[16] Shelokar P. S., Jayaraman V. K., Kulkarni B. D. (2004). An ant colony approach for clustering. *Analytica Chimica Acta*.

[17] Niknam T., Amiri B. (2010). An efficient hybrid approach based on PSO, ACO and k-means for cluster analysis. *Applied Soft Computing Journal*.

[18] Păun G. (2000). Computing with membranes. *Journal of Computer and System Sciences*.

[19] Martin-Vide C., Păun G., Pazos J., Rodriguez-Patón A. (2003). Tissue P systems. *Theoretical Computer Science*.

[20] Ionescu M., Păun G., Yokomori T. (2006). Spiking neural P systems. *Fundamenta Informaticae*.

[21] Păun G. (2001). P systems with active membranes attacking NP-complete problems. *Journal of Automata, Languages and Combinatorics*.

[22] Pan L., Ishdorj T. (2004). P systems with active membranes and separation rules. *Journal of Universal Computer Science*.

[23] Păun G., Pérez-Jiménez M. J., Riscos-Núñez A. (2008). Tissue P systems with cell division. *International Journal of Computers, Communications and Control*.

[24] Pan L., Pérez-Jiménez M. J. (2010). Computational complexity of tissue-like P systems. *Journal of Complexity*.

[25] Pan L., Pǎun G. (2009). Spiking neural P systems with anti-spikes. *International Journal of Computers, Communications and Control*.

[26] Pan L., Pǎun G., Pérez-Jiménez M. J. (2011). Spiking neural P systems with neuron division and budding. *Science China*.

[27] Wang J., Zou L., Peng H., Zhang G. (2011). An extended spiking neural P system for fuzzy knowledge representation. *International Journal of Innovative Computing, Information and Control*.

[28] Peng H., Wang J., Pérez-Jiménez M. J., Wang H., Shao J., Wang T. (2013). Fuzzy reasoning spiking neural P system for fault diagnosis. *Information Sciences*.

[29] Wang J., Shi P., Peng H., Perez-Jimenez M. J., Wang T. (2013). Weighted fuzzy spiking neural P systems. *IEEE Transactions on Fuzzy Systems*.

[30] Wang J., Peng H. (2013). Adaptive fuzzy spiking neural P systems for fuzzy inference and learning. *International Journal of Computer Mathematics*.

[31] Păun G., Rozenberg G., Salomaa A. (2010). *The Oxford Handbook of Membrane Computing*.

[32] Pǎun G., Pérez-Jiménez M. J. (2006). Membrane computing: brief introduction, recent results and applications. *BioSystems*.

[33] Alhazov A., Martín-Vide C., Pan L. (2003). Solving a PSPACE-complete problem by recognizing P systems with restricted active membranes. *Fundamenta Informaticae*.

[34] Ishdorj T., Leporati A., Pan L., Zeng X., Zhang X. (2010). Deterministic solutions to {\tt {QSAT}} and {\tt Q3{SAT}} by spiking neural P systems with pre-computed resources. *Theoretical Computer Science*.

[35] Zhang G., Cheng J., Gheorghe M., Meng Q. (2013). A hybrid approach based on differential evolution and tissue membrane systems for solving constrained manufacturing parameter optimization problems. *Applied Soft Computing Journal*.

[36] Peng H., Wang J., Pérez-Jiménez M. J., Shi P. (2013). A novel image thresholding method based on membrane computing and fuzzy entropy. *Journal of Intelligent and Fuzzy Systems*.

[37] Peng H., Wang J., Pérez-Jiménez M. J., Riscos-Núñez A. (2014). The framework of P systems applied to solve optimal watermarking problem. *Signal Processing*.

[38] Falkenauer E. (1998). *Genetic Algorithms and Grouping Problems*.

[39] Davis L. (1991). *Handbook of Genetic Algorithms*.

[40] http://www.ics.uci.edu/~mlearn/MLRepository.html.

